# Application of Manure Rather Than Plant-Origin Organic Fertilizers Alters the Fungal Community in Continuous Cropping Tobacco Soil

**DOI:** 10.3389/fmicb.2022.818956

**Published:** 2022-04-19

**Authors:** Yan Su, Haiyun Zi, Xiaomeng Wei, Binbin Hu, Xiaopeng Deng, Yi Chen, Yonglei Jiang

**Affiliations:** ^1^Yunnan Academy of Tobacco Agricultural Sciences, Kunming, China; ^2^Institute of Mountain Hazards and Environment, Chinese Academy of Sciences, Chengdu, China; ^3^College of Biology and the Environment, Nanjing Forestry University, Nanjing, China; ^4^Key Laboratory of Agro-Ecological Processes in Subtropical Region and Changsha Research Station for Agricultural and Environmental Monitoring, Institute of Subtropical Agriculture, Chinese Academy of Sciences, Changsha, China

**Keywords:** organic fertilizer, manure, fungal community, continuous cropping, tobacco

## Abstract

Continuous cropping leads to the development of serious fungal diseases in tobacco plants and depleted yield of tobacco (*Nicotiana tabacum*), which can be mitigated by organic fertilization. Yet, we know little about how organic fertilizers affect the fungal community of continuous cropping tobacco soil. In this study, we investigated the soil fungal community after 11 years of tobacco planting with chemical fertilization (CF) or chemical fertilization combined with organic fertilizers obtained from plant or animal origin, including oil cake (CFO), straw (CFS), and farmyard fertilizer (CFM). The predominant phyla of Ascomycota (70%) and Mortierellomycota (15%) were identified in all the treatments. A significantly higher proportion of Pyrenochaetopsis and lower relative abundance of Sordariomycetes were observed in the CFM group compared to the controls. Compared to CF and non-fertilized control (CK), CFO and CFS led to higher species richness (*P* < 0.05), while CFM led to a less uniform fungal community, indicated by lower Shannon and higher Simpson diversity indices (*P* < 0.05). Pearson’s correlation and redundancy analysis suggested that fertilizations primarily influenced the fungal community by altering the soil nutrient conditions, among which soil organic carbon and total phosphorus significantly correlated with the fungal diversity and community composition (*P* < 0.05). Notably, FUNGuild annotation suggested that while other treatments showed no significant effect on the fungal trophic modes, CFM strongly increased the abundance of saprotrophic fungi by more than 30% (*P* < 0.05), thus preventing the prevalence of potential pathotypes and symbionts. The results suggest that the type of organic fertilizers is essential to the long-term effects of organic application on the fungal community, and the animal-origin manure seems to be a better choice than plant-origin materials in continuous cropping tobacco fields.

## Introduction

Tobacco is one of the most important industrial crops that is intolerant to continuous cultivation (Chen et al., 2018). Even short-term continuous cropping would lead to a 20–100% decrease in the yield of tobacco, accompanied by impaired cigarette quality (Chen et al., 2015). Crop rotation has well-proven to improve the profitability of tobacco agriculture (Canalli et al., 2020); however, continuous cultivation is more commonly practiced due to the decrease of arable lands in the past decades. The mechanisms generating these obstacles remain merely revealed until now (Chen et al., 2020; Tan et al., 2021). Nevertheless, the essential role of the imbalance in the soil microbial community, in particular, the enriched fungal abundance and the increased ratio of fungi to bacteria, is generally accepted (Chen et al., 2018; Hao et al., 2021). About half of the tobacco diseases are caused by fungal pathogens, for example, *Phytophthora parasitica*, *Alternaria laternata*, and *Cercospora nicotianae*, and their population increases during continuous cropping (Wang et al., 2015). Thus, a better understanding of the fungal community in continuous cropping tobacco soil is necessary, which is, however, little studied.

Organic fertilization is known to mitigate the damage caused by continuous cultivation on tobacco production (Dai et al., 2021; Wang et al., 2022). A decrease in soil pH is fundamentally responsible for the prevalence of fungi in continuous cropping soil, which is aggravated by the intensive application of mineral nitrogen (Guo et al., 2010; Behnke et al., 2020). The negative surface charges of organic matter provide a neutral power to consume the proton derived from ammonia nitrification. As widely acknowledged, the introduction of organic fertilizers dramatically alleviates the soil acidification process in multiple continuous cropping systems, including tobacco, which would reshape the soil fungal community (Dai et al., 2021; Shen et al., 2021). Additionally, the fungal community in the soil is tightly regulated by the nutrient contents. Previous reports indicated that the boost of fungal pathogens in the continuous cropping field was likely due to the depletion of organic C and the increase in N content, which was reversed by organic fertilization (Aguilar et al., 2017). Despite these findings, the effect of organic fertilization on the fungal community in continuously cultivated tobacco soil has never been investigated to our best knowledge.

The regulatory effect of organic fertilization on the soil fungal community depends on the properties of organic fertilizer, which are determined by the production materials (Tian et al., 2021). Current organic fertilizers are generally produced using plant residues or animal excrements, which exhibit remarkably different chemical and biological characteristics. For instance, compared to plant-derived organic fertilizers, manure-derived organic fertilizers are abundant in N and P nutrients and contain more living microorganisms (Xia et al., 2021). As a result, the response of the soil fungal community to organic fertilizers varied with the fertilizer type (Ding et al., 2017; Llimos et al., 2021). The differences reflect in the diversity and species composition of the community as well as the tropic models (Wang et al., 2018). As previously reported, the combined application of manure and chemical fertilizer is frequently reported to decrease the relative abundance of potential pathographic fungi, and the application of straw increases the frequency of pathogens of rhizosphere soil in many cases (Glen-Karolczyk et al., 2018; Fan et al., 2020; Wen et al., 2020). Nevertheless, this problem is scantly addressed in the tobacco field.

In this study, using the Illumina (Novaseq 6000) sequencing of the internal transcribed spacer (ITS) region, we investigated the soil fungal community after 11 years of consecutive tobacco cropping with the application of chemical fertilizers or three different organic fertilizers. The objectives of our study were (1) to assess the effect of long-term application of different organic fertilizers on the fungal community of tobacco cultivation soil of continuous cropping field and (2) to reveal the correlation between soil physicochemical properties and fungal community composition. We hypothesized that (1) the application of organic matter combined with chemical N would increase the fungal diversity and alter the fungal trophic model to a guild with fewer potential pathogens and that (2) the manure application would have a stronger effect on the fugal community than other organic fertilizers. This study is one of the earliest documents to detail the fungal community of tobacco soil with continuous cropping and its responses to fertilization management. Our findings would help to alleviate the continuous cropping obstacles in tobacco systems and to supply positive management strategies for policymakers and farmers.

## Materials and Methods

### Experiment Setup and Soil Sampling

The field experiment was conducted from 2006 to 2016 in Yanhe Experimental Base in Yanhe Town, Yuxi City, Yunnan Province (N 24°14′, E 102°30′), at an altitude of 1,680 m. The annual average temperature and sunshine duration are 15.9°C and 2,072 h, respectively, and the annual average precipitation is 918 mm, over 79.5% of which occurs in the “wet season” between April and September (Wang et al., 2022). The soil type is typical sandy red soil with 28% sandy, 50% loam, and 22% clay. The properties of the top soil (0–20 cm) are as follows: organic matter, 10.70 g kg^–1^; total nitrogen (TN), 0.54 g kg^–1^; total phosphorus (TP), 0.11 g kg^–1^; total potassium (TK), 6.43 g kg^–1^; available N, 82.0 mg kg^–1^; available P, 9.01 mg kg^–1^; available K, 160.0 mg kg^–1^; and pH 6.4.

A randomized complete block design was used in this study. Briefly, 15 plots were marked (28 m^2^, 14 m × 2 m) in the experimental site, and each plot was separated by a 0.3-m-wide buffer strip. Five fertilization treatments were randomly applied to the plots in triplicate: (i) no fertilizer application (CK); (ii) chemical N fertilizer, 75 kg N ha^–1^ (CF); (iii) 450 kg oil cake ha^–1^ + 75 kg N ha^–1^ (CFO); (iv) 3,000 kg straw ha^–1^ + 75 kg N ha^–1^ (CFS); and (v) 15,000 kg farmyard fertilizer (pig manure) ha^–1^ + 75 kg N ha^–1^ (CFM). Tobacco (*Nicotiana tabacum* L. “K326”) was transplanted in May and harvested in August each year. Before the transplantation of tobacco seedlings, 50% of chemical N and all organic fertilizers were applied, and the remaining chemical N was applied 7 and 20 days after the transplantation, 25% each time. Immediately after the transplantation, the seedlings were trickle irrigated with 200-time diluted cuaminosulfate aqueous solution (14%) and 1,000-time diluted Fubol wettable powder (58%). To further inhibit the development of diseases, a second trickle irrigation with 1,000 times diluted Fubol wettable powder (58%) was applied 15 days after transplantation, and 500 times diluted Fubol wettable powder (58%) was sprayed for two times after 35 and 50 days of transplanting the seedlings.

Soil samples (0–20 cm) were collected in October 2016. Five soil cores (0–20 cm depth, 5 cm diameter) were randomly collected from each plot and mixed as a composite soil sample after the tobacco was harvested. A total of 15 soil samples were obtained (three replicates × five treatments). The samples were placed in an icebox and sent to the laboratory immediately. Then, the samples were filtered through a 2-mm sieve to remove roots, rocks, and litter. The sieved samples were divided into three subsamples. One portion of the sample was stored at -80°C for soil microbial community assessment, and the second one was stored at 4°C for the determination of soil physicochemical properties. The third subsample was air-dried and passed through a 100-mesh (0.15 mm) sieve for the determination of soil organic carbon (SOC) concentration.

### Soil Biogeochemical Analysis

A glass combination electrode immersed in a suspension of soil and water at a ratio of 1: 2.5 (w: v) was used to determine the soil pH. Soil electronic conductivity (EC) was measured using a conductivity meter (2.5: 1, water/soil ratio). Soil organic carbon (SOC) was determined by the dichromate oxidation method (Kalembasa and Jenkinson, 1973). Soil total nitrogen (TN) was determined using a semi-automatic Kjeldahl Azotometer (Alva, China) and total phosphorus (TP) by the vanado-molybdate phosphoric yellow colorimetric procedure (Bao, 2000). Total potassium (TK) was determined using atomic absorption spectrophotometry (AA-6800, Shimadzu, Japan) (Jackson, 1973). A 0.5 M K_2_SO_4_ solution was used to extract soil NH_4_^+^-N and NO_3_^–^-N, and their concentrations in the supernatant were determined in a San^++^ Continuous Flow analyzer after filtering through a 0.45-μm membrane (Skalar, Netherlands).

### DNA Extraction, Polymerase Chain Reaction Amplification, Illumina MiSeq Sequencing, and Data Processing

The microbial DNA was extracted from 0.5 g of soil samples using a Fast DNA SPIN kit (MP Biomedicals, CA, United States) according to the manufacturer’s instructions. NanoDrop ND-1000 spectrophotometer was used (NanoDrop Technologies Inc., Wilmington, DE, United States) to determine the quality and quantity of DNA. The A260/A280 values of all the DNA extractions were in the range of 1.81–1.95, and A260/A230 values were higher than 1, which well-met the quality required for sequencing. The DNA quality was further verified using 1% agarose gel electrophoresis and was stored at -80°C.

A primer pair consisting of ITS1F (5′-CTTGGTCATTTA GAGGAAGTAA-3′) and ITS2R (5′-GCTGCGTTCTTCATCG ATGC-3′) primers were used to amplify the fungal ITS rDNA genes (White et al., 1989). The PCR reaction was carried out in a solution containing 20 ml of mixture buffer, 2 μl of 2.5 mM deoxynucleotide (dNTPs), 0.8 μl of each primer (5 μM), 0.4 ml of FastPfu Polymerase, 0.2 μl of bovine serum albumin (BSA), and 10 ng of DNA template. The PCR reactions were conducted according to the following program: denaturation for 3 min at 95°C; 35 cycles of 30 s each at 95°C, annealing at 55°C for 30 s, and extension at 72°C for 45 s; and finally, extension at 72°C for 10 min. The PCR products were extracted by 2% agarose gel electrophoresis, purified using an AxyPrep DNA Gel Extraction Kit (Axygen Biosciences, CA, United States), and quantified using QuantiFluor™-ST (Promega, Madison, WI, United States), in accordance with the manufacturer’s instructions. The purified amplicons were paired-end sequenced (2 × 300) on an Illumina MiSeq platform (Illumina, San Diego, CA, United States) by Majorbio Bio-Pharm Technology Co. Ltd. (Shanghai, China). The paired-end sequencing data were merged by using the FLASH program, version 1.2.10^1^ and filtered by using Trimmomatic software (Magoc and Salzberg, 2011; Edgar, 2013). Raw data that contained an N base or had an average quality score <20 and sequence length <50 bp were discarded. In total, 781,250 high-quality reads were obtained from all the samples. The sequencing depth was rarefied to 45,000 reads per sample and imported to UPARSE software for the clustering of operational taxonomic units (OTUs) at the similarity cutoff of 0.97 (Edgar, 2013). For each OTU, the most abundant sequence was selected as the representative sequence and was taxonomically classified using BLAST against the UNITE reference database, and a BLASTN search was performed to assign the OTU classification at a confidence threshold of 80% (Abarenkov et al., 2010). Using the “diversity” function in the *vegan* package (Oksanem et al., 2013) in R (version 3.3.2, R Core Team, 2015), alpha diversity indices, such as the observed OTUs, Chao1, Shannon index, and Simpson index, were calculated, where the former two indicated the species richness while the latter two provided the evenness information of the community. The raw sequence data reported in this study have been deposited in the Genome Sequence Archive (Genomics, Proteomics, and Bioinformatics 2017) of the National Genomics Data Center (Nucleic Acids Res 2021), China National Center for Bioinformation/Beijing Institute of Genomics, Chinese Academy of Sciences, under the accession number CRA003510, and the data are publicly accessible at https://ngdc.cncb.ac.cn/gsa.

### Fungal Trophic Modes

In the fungal community ecology, there are three trophic modes: pathotrophs, symbiotrophs, and saprotrophs. Each OTU was classified according to these trophic modes by using the online FUNGuild database,^2^ a tool that estimates the potential function of fungal taxa based on the summary of literature studies (Nguyen et al., 2015). Similar to the previous studies (Veach et al., 2017), only guild assignments with “highly probable” and “probable” confidence levels were retained for the next statistical analysis. The OTUs that do not match the taxa mentioned in the database were categorized as “unassigned” and were not included in the interpretation of our results. The relative abundance was estimated by dividing the total reads by the reads for each functional group under each experimental treatment.

### Statistical Analysis

A one-way analysis of variance (ANOVA), followed by Tukey’s test at *P* < 0.05 for each of the variables, was used to examine the differences between the different treatments using SPSS 16.0 (SPSS Inc., Chicago, IL, United States). The relationship between soil chemical properties and fungal diversities and compositions was identified by Spearman’s correlation analysis using SPSS 16.0 (SPSS Inc., Chicago, IL, United States). The beta diversity was calculated by performing the non-metric multidimensional scaling analysis (NMDS) based on the unweighted UniFrac distance between all the 15 samples using the *metaMDS* function in the *vegan* package in R (R 3.6.2). The distance matrix was transformed by performing the Wisconsin double standardization. Significant differences between the treatments were analyzed using the *adonis* function in the *vegan* package (R 3.6.2). We performed LEfSe using the Galaxy pipeline^3^ to determine the species biomarker in our soil samples. OTUs that showed significantly different abundance levels between the treatments and an absolute linear discriminant analysis (LDA) value higher than two were described as the indicator species (*P* < 0.05). The relationship between soil properties and microbial community composition was assessed by redundancy analysis (RDA) using CANOCO version 5.0 for Windows (Microcomputer Power, Ithaca, NY, United States), followed by Monte Carlo permutations (calculated based on 999).

## Results and Discussion

### Soil Properties

The results presented in Table 1 show that organic fertilization (CFO, CFM, and CFS) mitigated the acidification of tobacco soil when compared to the treatment applied with mono-chemical fertilizers (CF) (*p* < 0.05). However, no significant differences were found between the three treatments with different organic fertilizers. Higher SOC and TN levels were observed in the organically fertilized soil when compared to the CF content, but the difference was significant only for CFM (*p* < 0.05). Furthermore, CFM led to the highest SOC, TN, NO_3_^–^, TP, and EC levels, while CFO markedly increased NH_4_^+^ ion concentration when compared to the other fertilization regimes (*p* < 0.05). On the contrary, all the tested soil chemical properties were comparable between CF and CK treatments, except for the lower pH value and higher NO_3_^–^ concentration (*p* < 0.05).

**TABLE 1:** Soil physicochemical properties after the long-term application of different fertilizers.

Treatment	pH	SOC (g kg^–1^)	TN (g kg^–1^)	NH_4_^+^ (mg kg^–1^)	NO_3_^–^ (mg kg^–1^)	TP (g kg^–1^)	TK (g kg^–1^)	EC (μ s cm^–1^)
CFO	7.03 ± 0.23a	6.88 ± 0.47b	0.76 ± 0.12b	2.58 ± 0.87a	35.10 ± 5.43b	1.02 ± 0.07b	6.12 ± 0.25a	143.80 ± 64.48ab
CFM	7.01 ± 0.20a	8.87 ± 0.65a	0.97 ± 0.12a	1.34 ± 0.57b	81.20 ± 8.51a	1.31 ± 0.08a	6.14 ± 0.20a	222.67 ± 112.06a
CFS	7.02 ± 0.08a	6.57 ± 0.27bc	0.78 ± 0.06b	0.20 ± 0.01c	20.06 ± 2.10c	1.03 ± 0.08b	5.97 ± 0.36a	157.50 ± 10.78ab
CF	6.66 ± 0.11b	6.21 ± 0.10bc	0.55 ± 0.05c	0.37 ± 0.07c	37.79 ± 13.94b	0.97 ± 0.13b	5.64 ± 0.75a	124.37 ± 54.16ab
CK	7.30 ± 0.16a	5.85 ± 0.45c	0.58 ± 0.12c	0.56 ± 0.47bc	17.17 ± 5.81c	0.90 ± 0.07b	5.98 ± 0.34a	85.23 ± 12.20b

*The data presented in the tables are expressed as mean ± standard error values (n = 3). Different lowercase letters indicate significant difference at P < 0.05 (Tukey’s test). SOC, soil organic carbon; TN, total nitrogen; NH_4_^+^, Ammonia nitrogen; NO_3_^–^, Nitrate nitrogen; TP, total phosphorus; TK, total potassium; EC, electrical conductivity.*

Long-term continuous cropping generally led to a decline in the soil nutrients (Miao et al., 2011). However, the application of chemical fertilizers facilitates the replenishment of lost nutrients, consequently improving plant growth and yield (Smith et al., 2016). In this study, the composition of none of the soil nutrients (except NO_3_^–^) was significantly influenced by the addition of chemical fertilizers when compared to that observed in the CK group (Table 1). The results indicated that most of the nutrients present in the chemical fertilizers were exhausted during the cropping season, as a result of the plant uptake, leaching, and gas loss (Wang and Li, 2019; Kaur et al., 2020). Therefore, the mono-application of chemical fertilizers did not actually improve the soil fertility in the continuous cropping tobacco fields. This observation is common in multiple cropping systems, and the combination of chemical and organic fertilizers has been proposed to solve this problem (Miao et al., 2011; Chen et al., 2022). As reported by Wei et al. (2016), the soil productivity increased by an average of approximately 20% when 32 long-term fertilization experiments were conducted by combining organic and chemical fertilizers. Similarly, the application of all three types of organic matter led to a higher content of C, N, and P compared to that observed in the CK and CF groups in this study; however, only the effect of CFM was found to be significant in all the treatment regimes (Table 1). Our data suggested that the animal-derived manure was more efficient in improving the soil nutrients during the continuous cropping of tobacco.

### Diversity of Fungal Community

**FIGURE 1:**
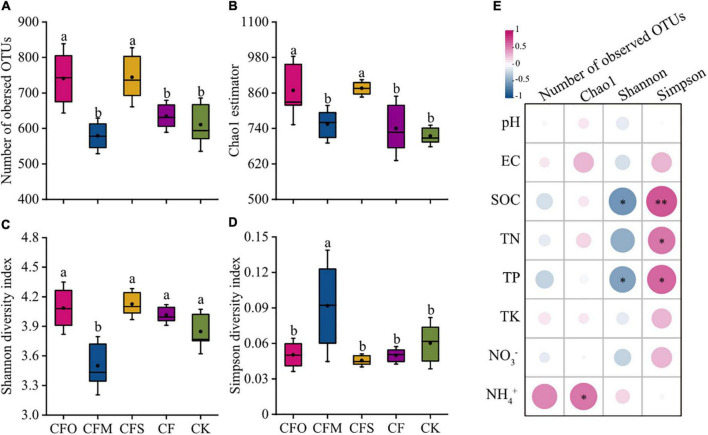
Fungal alpha diversity index values observed in different fertilization management systems in continuous tobacco cropping soils **(A–D)**; Pearson’s correlation analysis between alpha diversity index and soil parameters **(E)**. Different lowercase letters indicate significant differences at *P* < 0.05 (Tukey’s test). SOC, soil organic carbon; TN, total nitrogen; TP, total phosphorus; TK, total potassium; EC, electrical conductivity. The significant levels are indicated by asterisk: *p < 0.05; ^**^p < 0.001.

**FIGURE 2:**
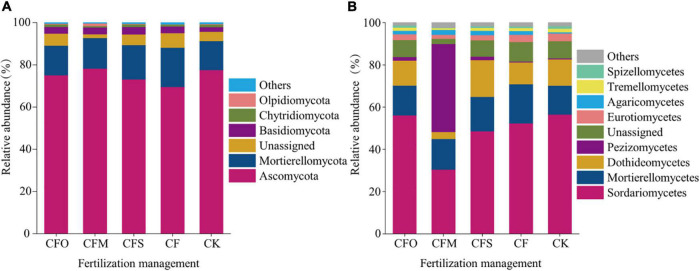
The relative abundance of the dominant fungal phyla **(A)** and classes **(B)** under different fertilization management systems in continuous tobacco cropping soils.

Mono-chemical fertilization showed little effect on the fungal alpha diversity index when compared to the CK group (Figures 1A–D). However, CFO and CFS treatments significantly increased the richness of fungal species, which were indicated by more number of observed OTUs and higher Chao1 index (Figures 1A,B, *P* < 0.05). By contrast, manure application did not affect the OTU number and the Chao1 index of the fungal community, whereas it led to a higher Simpson index and a lower Shannon index (Figures 1C,D), which indicated a decay in the species evenness. It can be inferred from the consistent species richness but decreasing diversity that a few dominant species were enriched in CFM, for example, *Pezizomycetes* (Figure 2B), while a large number of rare species also existed. Although the previous reports indicated that manure fertilization improved the soil fungal diversity by providing available nutrients abundantly (Ding et al., 2017), we found a positive correlation between SOC, TN, and TP levels and the Simpson index and a negative correlation with the Shannon index (Figure 1E), which indicated that the fungal diversity depleted in the presence of increased soil nutrients in this study. The resource competition theory suggests that the microbial diversity is the highest under moderate resource limitation, while it depleted when the environmental resources were limited or superfluous, due to competitive exclusion or favoring the growth of eutrophic species (Graham and Duda, 2011; Wei et al., 2021). The results likely suggest that the long-term application of manure resulted in excessive available C and nutrients in our tobacco soil, which could be supported by the soil properties, reported earlier (Table 1).

**FIGURE 3:**
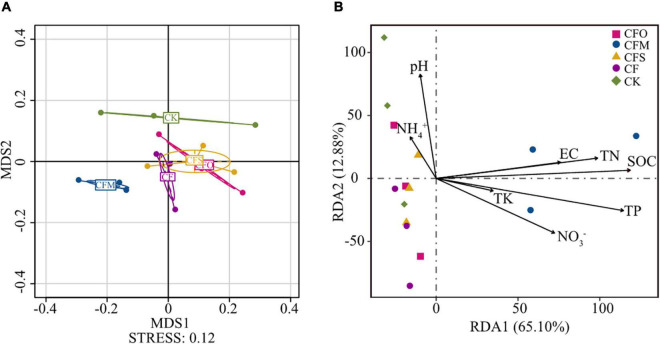
Fungal community structure in different fertilization regimes showed by a non-metric multidimensional scaling (NMDS) plot **(A)**. Redundancy analysis (RDA) of soil physicochemical properties with soil fungal community structure **(B)**. SOC, soil organic carbon; TN, total nitrogen; TP, total phosphorus; TK, total potassium; EC, electrical conductivity.

The NMDS plots showed that the soil samples subjected to different treatments could be divided into four groups (Figure 3A). While the fungal community in CMF was most distant from CK, the chemical fertilization (CF) and chemical fertilization combined with oil cake (CFO) or straw (CFS) moderately affected the fungal community (*p* < 0.05, Figure 3A and Supplementary Table 1). In comparison to CF, the application of all three types of organic matter significantly influenced the fungal community of tobacco soil (*p* < 0.05, Supplementary Table 1); nevertheless, the shifts caused by CFO and CFS were of much slighter intensity than CFM (Figure 3A), which was consistent with previous studies (Ma et al., 2018). It was reported that the continuous long-term application of organic and nitrogen fertilizer significantly affected the fungal community composition by changing the soil nutrient availability (Gu et al., 2019). In our study, the results of the RDA analysis indicated that SOC was the primary regulator that affected the fungal community, followed by TP and TN (Figure 3B). It is no surprise that the C resource played a key role in determining the fugal community composition, since fungi are totally heterotrophic biota (Loganathachetti et al., 2017). Nitrogen is the second major element found in the body cells and intrinsically influences the microbial community (Widdig et al., 2020). However, P is important in controlling the immune responses of the plant to escape from the soil microbial assembly and resistant pathogenic organisms (Zhao et al., 2020). This finding may explain the importance of P over N in this study because continuous cropping leads to an increasing crisis of fungal diseases in the tobacco plants. Consistent with our study results, similar results were previously reported (Jin et al., 2021; Sun et al., 2021). Previous studies also suggested pH to be the most important factor that impacts the fungal community (Zhou et al., 2016; Tarin et al., 2021). However, its effect was negligible in our study (Figure 3A), probably because the pH values of all the soils were nearly neutral (Table 1).

### Community Composition and Indicator Species

The results showed that *Ascomycota* and *Mortierellomycota* (13.7–18.6%) were the dominant phyla in the fungal community, accounting for more than 80% of the fungal community (Figure 2A). *Ascomycota* members were the most abundant in CFM treatment (78.2%) and the least abundant in CF treatment (69.6%). CFM treatment led to the relative abundance of *Ascomycota*, which increased by 0.62–8.67%, compared to that observed in the other four treatments (CK, CF, CFO, and CFS). At the class level, the dominant fungi (relative abundance >10%) mainly belonged to *Sordariomycetes* and *Mortierellomycetes* (Figure 2B). CFM treatment resulted in a decreased population of *Sordariomycetes* and unassigned fungi but increased the relative abundance of *Pezizomycetes* fungi relative to the other four treatments. The CFM treatments increased the relative abundance of the members of class *Pezizomycetes* by 40–41.2% compared to that noticed after CFO, CFS, CF, and CK treatments (Figure 2B).

A total of 140 fungal OTUs were selected from all the samples as the indicator species for analysis (*P* < 0.05). Most of the indicator species belonged to *Ascomycota*, followed by *Mortierellomycota*, *Basidiomycota*, and *Chytridiomycota* (Supplementary Figure 1). At the genus level, there were significant differences in the indicator species, with indicator values higher than 0.75 in different fertilization management groups. The indicator species in the CFO group were identified as *Cephalotrichum, Chaetomium*, and *Latorua*. The indicator species in the CFM group were identified as *Acaulium, Aphanoascus, Arachniotus, Cephaliophora*, and *Chrysosporium*, *Bipolaris, Exophiala*, and *Olpidium* were more abundant in CFS soils. *Geomyces* were more abundant in the CF soils, while *Acremonium*, *Fusicolla*, and *Aspergillus* were more abundant in the CK soils (Supplementary Figure 1). Among the five fertilization management groups, more number of indicator species were found in the CFM group, but the number was less in the CK group.

Different fertilization management strategies altered the soil properties, leading to a shift in the species composition and diversity of the fungal community (Ullah et al., 2019). In this study, we found that *Ascomycota* and *Mortierellomycota* were the dominant phyla across all the different fertilization regimes (Figure 2A), which was in line with the results of a previous study conducted in continuous maize cropping soils (Zhao et al., 2021). *Ascomycota*, which are typical decomposers (Ma et al., 2013), is the predominant taxa in agricultural soils (Rousk et al., 2010; Egidi et al., 2019; Tang et al., 2020). At the class level, *Sordariomycetes* was the most dominant class across the treatments, except in the CFM group. The farmyard fertilizer combined with chemical fertilizer significantly increased the relative abundance of class *Pezizomycetes* and decreased the abundance of class *Sordariomycetes* and *Dothideomycetes*, compared to the abundance observed in the other four treatments (Figure 2B), which was contradictory to the previous findings of Ding et al. (2017) who reported that the incorporation of inorganic fertilizer and manure significantly increased the abundance of *Sordariomycetes* in northeastern China. At the genus level, more number of indicator species were found in the CFM group and were identified as *Acaulium*, *Aphanoascus*, *Arachniotus*, *Cephaliophora*, and *Chrysosporium*, which indicated that combining pig manure with N fertilizer influenced the fungal composition and changed the dominant genera.

### Fungal Trophic Modes

**FIGURE 4:**
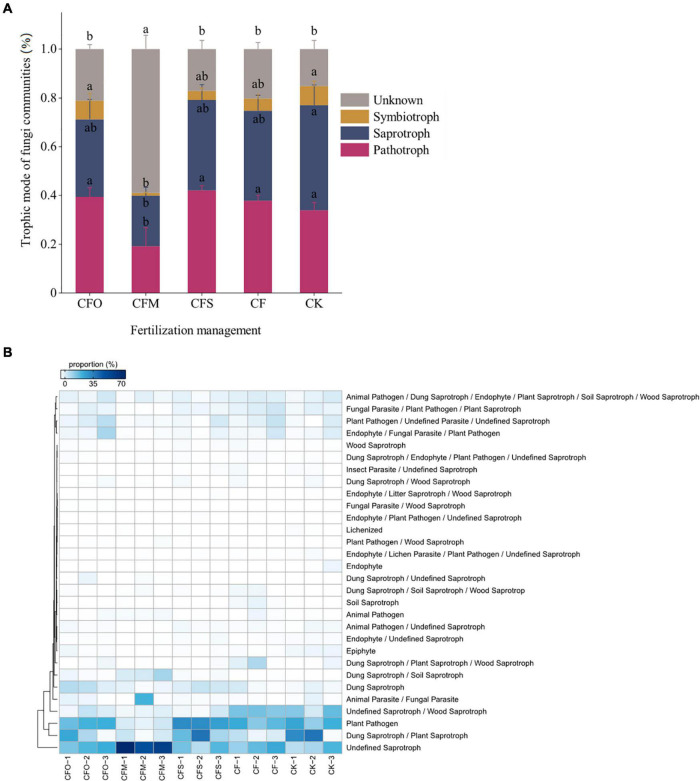
Fungal trophic modes **(A)** and the details of each trophic medels **(B)** in tobacco continuously mono-cropped soils under different fertilization management systems. Different lowercase letters indicate significant differences at *p* < 0.05 (Tukey’s test).

The application of fertilizers significantly altered the trophic modes of the fungal community in continuous cropping tobacco soil when compared to that observed in the CK group (*P* < 0.05; Figure 4). The structure of fungal trophic modes in the CK group was as follows: 43% saprotrophs + 34% pathotrophs + 8% symbiotrophs + 15% unknown trophic modes (Figure 4A). Compared to the CK group, a decrease in the saprotrophs by 6% and an increase in the pathotrophs by 4% were noticed in the CF group. CFM treatment significantly decreased the pathotrophs and symbiotrophs but increased the unknown fungi compared to other treatments. The abundance of pathotrophs was the highest in the CFS treatment, while it was the lowest in the CFM treatment (Figure 4A). More than 50% of the unidentified saprotrophs were found in the CFM treatment, while in other treatments, the proportion was approximately 20% (Figures 4A,B). The results indicated that the functions of most of the fungi observed in the CFM group were little studied, much less than the fungi identified in other treatment groups. However, as the pathogens are generally specific, expansively studied, and well-assigned, these OTUs are most unlikely to represent any known pathogen. As manure application was found to decrease the incidence of fungal diseases, we suppose that these OTUs potentially inhibited the growth of pathogens, and these assumptions need to be proved by further studies.

Pathotrophic fungi exhibit a negative impact on plant growth by damaging the host cells to obtain nutrients (Tedersoo et al., 2014; Xie et al., 2020), and the saprotrophic fungi are the primary decomposers in the soil (Thormann, 2006; Xie et al., 2020). In this study, CF, CFO, and CFS treatments reduced saprotrophs but increased pathotrophs, which would increase the incidence of diseases and thus affect plant growth (Nie et al., 2018). Conversely, the application of CFM significantly reduced the relative abundance of pathotrophic fungi and increased the saprotrophs, which might restrain soil-borne fungal pathogens (Wang et al., 2018). The SOC in CFM was 30% higher than that observed in other treatments, which indicated an indiscriminate increase in the biomass of both pathogens and non-pathogens (Wang et al., 2018). Nevertheless, higher relative abundance of saprophytic fungi indicated that the parasitic guilds were less competitive in the fungal community, which is more frequently detected as plant pathogens (Liddicoat et al., 2018). Other studies have also shown that the application of organic manure reduced the relative abundance of soil-borne fungal pathogens (Wen et al., 2020). A probable reason is that the application of organic manure can improve the ventilation conditions of the soil and stimulate beneficial microbes (Ding et al., 2017; Wen et al., 2020). It is intriguing that most of the saprotrophs in CFM are undefined, which might due to the differences in the compounds of manure and plant-origin materials (Li et al., 2021). This may also be an explanation for the decreased effects of CFO and CFS on the fungal community than the effect showed by CFM, since the C sources provided by manure were thoroughly different from the C of living plants and plant residues. Nevertheless, the mechanism of soil microbial function in long-term fertilization regimes in continuous cropping tobacco soil needs further study, especially to reveal the roles of these undefined saprotrophic fungi.

## Conclusion

Long-term organic fertilization led to a significantly different fungal community compared to non-fertilized and chemically fertilized soils; however, the exact effect depended on the type of organic fertilizer. Compared to the plant-derived organic fertilizers (oil cake and straw), long-term manure application decreased the fungal diversity and the relative abundance of pathotrophic species. A correlation analysis with the soil chemical properties indicated that the results were likely due to the excessive labile resource from manure. In conclusion, we suggest that manure would be a better organic fertilizer than oil cake and straw in a continuous cropping tobacco field.

## Data Availability Statement

The datasets presented in this study can be found in online repositories. The names of the repository/repositories and accession number(s) can be found in the article/Supplementary Material.

## Author Contributions

YS and HZ contributed to the study design, data collection, and manuscript writing. YS, XW, YC, BH, and XD performed the experimental measurements and contributed to manuscript writing. YJ supervised the study and contributed to manuscript writing. All authors read and approved the final manuscript.

## Conflict of Interest

The authors declare that the research was conducted in the absence of any commercial or financial relationships that could be construed as a potential conflict of interest.

## Publisher’s Note

All claims expressed in this article are solely those of the authors and do not necessarily represent those of their affiliated organizations, or those of the publisher, the editors and the reviewers. Any product that may be evaluated in this article, or claim that may be made by its manufacturer, is not guaranteed or endorsed by the publisher.

## Footnotes

1
https://ccb.jhu.edu/software/FLASH/

2
https://github.com/UMNFuN/FUNGuild

3
http://huttenhower.sph.harvard.edu/galaxy

## Supplementary Material

Supplementary Figure 1 Fungal genera were identified as indicator taxa significantly (*p* < 0.05) associated with different fertilization management systems in continuous tobacco cropping soils. The bars represent the cumulative relative abundance of each indicator’s operational taxonomic unit (OTUs). Each circle defines the association strength (indicator value), such as <0.25: not characteristic; 0.25–0.5: weakly characteristic; 0.5–0.75: characteristic; >0.75: strongly characteristic.
