# Supplementary material for: Application of Manure Rather Than Plant-Origin Organic Fertilizers Alters the Fungal Community in Continuous Cropping Tobacco Soil
Source: Front Microbiol. 2022 Apr 19;13:818956. doi: 10.3389/fmicb.2022.818956 (PMC9063659; doi:10.3389/fmicb.2022.818956)
Supplement: Supplementary file 1 [file Table_2.DOCX]

|  | F | *p* |
| --- | --- | --- |
| CFM vs CFO | 4.967638 | 0.03 |
| CFS vs CFO | 1.379173 | 0.34 |
| CFS vs CFM | 4.646507 | 0.03 |
| CF vs CFO | 4.291374 | 0.04 |
| CF vs CFM | 5.725058 | 0.02 |
| CF vs CFS | 4.53728 | 0.05 |
| CK vs CFO | 4.544607 | 0.05 |
| CK vs CFM | 5.41891 | 0.02 |
| CK vs CFS | 4.720375 | 0.03 |
| CK vs CF | 4.55475 | 0.04 |

Table S1 Permanova analysis showing the significant difference among fertilization treatments.
