# Supplementary figures and images for: Application of Manure Rather Than Plant-Origin Organic Fertilizers Alters the Fungal Community in Continuous Cropping Tobacco Soil
Source: Front Microbiol. 2022 Apr 19;13:818956. doi: 10.3389/fmicb.2022.818956 (PMC9063659; doi:10.3389/fmicb.2022.818956)

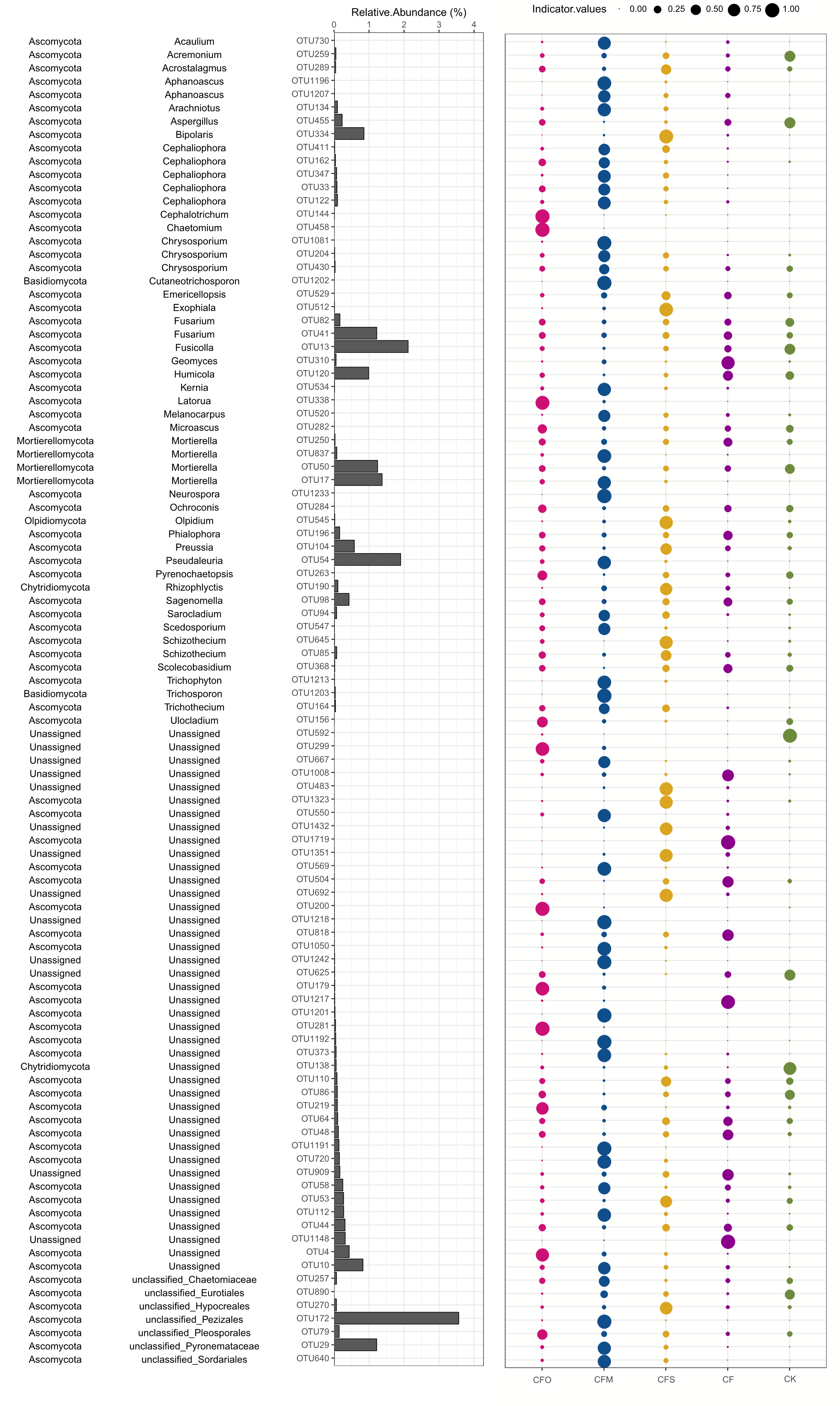

Supplement: Supplementary Figure 1 — Fungal genera were identified as indicator taxa significantly (p < 0.05) associated with different fertilization management systems in continuous tobacco cropping soils. The bars represent the cumulative relative abundance of each indicator’s operational taxonomic unit (OTUs). Each circle defines the association strength (indicator value), such as <0.25: not characteristic; 0.25–0.5: weakly characteristic; 0.5–0.75: characteristic; >0.75: strongly characteristic. [file Image_1.tiff]
